# The location of olfactory receptors within olfactory epithelium is independent of odorant volatility and solubility

**DOI:** 10.1186/1756-0500-4-137

**Published:** 2011-05-06

**Authors:** Tatjana Abaffy, Anthony R DeFazio

**Affiliations:** 1 Department of Molecular and Cellular Pharmacology, Miller School of Medicine, University of Miami, 1600 NW 10thAve, Miami, 33136, Fl, USA; 2 Department of Neurology, Miller School of Medicine, University of Miami,1420 NW 9thAve, Miami, 33136, Fl, USA

## Abstract

**Background:**

Our objective was to study the pattern of olfactory receptor expression within the dorsal and ventral regions of the mouse olfactory epithelium. We hypothesized that olfactory receptors were distributed based on the chemical properties of their ligands: e.g. receptors for polar, hydrophilic and weakly volatile odorants would be present in the dorsal region of olfactory epithelium; while receptors for non-polar, more volatile odorants would be distributed to the ventral region. To test our hypothesis, we used micro-transplantation of cilia-enriched plasma membranes derived from dorsal or ventral regions of the olfactory epithelium into Xenopus oocytes for electrophysiological characterization against a panel of 100 odorants.

**Findings:**

Odorants detected by ORs from the dorsal and ventral regions showed overlap in volatility and water solubility. We did not find evidence for a correlation between the solubility and volatility of odorants and the functional expression of olfactory receptors in the dorsal or ventral region of the olfactory epithelia.

**Conclusions:**

No simple clustering or relationship between chemical properties of odorants could be associated with the different regions of the olfactory epithelium. These results suggest that the location of ORs within the epithelium is not organized based on the physico-chemical properties of their ligands.

## Findings

The molecular events that lead to olfactory perception can be divided into peripheral (detection by olfactory receptors (ORs) in the nasal epithelium) and central (olfactory bulb and cortex). The events that occur at the peripheral level are not only represented by odorant-receptor affinity, but also include the physico-chemical characteristics of odorants, their diffusion through the mucus, air flow dynamics, as well as the spatial distribution of olfactory receptors within the olfactory epithelium [1-3]. The main olfactory system has a diverse population of receptors (for review see [4]). Most of these receptors remain orphans with no known ligand. Thus, the functional organization of the peripheral olfactory system remains theoretical, particularly in mammals.

Odorant discrimination is mediated by ORs using combinatorial coding: a single OR can be activated by multiple odorants and most odorants activate more than one OR [5,6]. Odorants represent a vast array of different chemical structures and each receptor samples a specific region of "chemical space" meaning that it is activated by one or a few combinations of chemical features [7]. A small change in the odorant molecule can result in a fundamental change of its molecular properties (such as functional group, length, flexibility, hydrophobicity, volatility, polarity, chemical bonds) and consequently may change or negate detection by a given OR.

In mammals, division of olfactory epithelium into dorsal and ventral regions is based on anatomical [8], biochemical [9,10] and behavioral [11] differences. Do these regions have different populations of receptors with distinct functional roles? Mouse olfactory receptors are divided into Class I and Class II receptors based on phylogenetic analysis [12]. Class I genes are the only type found in fish [13]. Both Class I and II ORs are found in amphibians and terrestrial vertebrates [14]. Classically, the olfactory epithelium has been divided along a dorso-ventral axis into four zones based on OR expression [15,16]. The dorsal region (also called Zone I) expresses about 50% of all OR genes, exclusively Class I and some of Class II receptors. The ventral region consists of endoturbinates II, III and IV and expresses only class II OR genes [17-20]. The dorsal region is exposed to near ambient concentrations of toxins and air pollutants. Thus, it is not surprising that this region is associated with high expression of antioxidant, chemo-protective enzymes: NADPH quinone oxido-reductase 1 (NQO1) [21], NADPH diaphorase [22], glutathione peroxidase, catalase and superoxide dismutase [23]. This region is also rich in complex glycolipids [24] and expresses an olfactory specific medium chain acyl-CoA synthetase (O-MACS, EC 6.2.1.2) [10]. Interestingly, mice lacked innate responses to aversive odorants after transgenic oblation of the dorsal zone using O-MACS driven expression of diphtheria toxin [11].

The ventral region of the olfactory epithelium has a complex turbinate structure, and as documented for hamster olfactory epithelium has three times more luminal surface area than the dorsal region [25,26]. The ventral region also expresses different transcription factors [27] and the olfactory-specific cell adhesion molecule OCAM, also known as NCAM2 [9,28,29].

Early experiments demonstrated that different odorants activate different regions of the olfactory epithelium [30-34]. It was hypothesized that two processes could be responsible for this topographic code: (1) the "inherent" patterning process, based on the idea that ORs with similar responses are grouped in similar regions of the epithelium and (2) the "imposed" patterning process, based on the morphology of the nasal cavity, on the pattern of airflow during sniffing, and on the differential adsorption of the odorants through the olfactory mucus [35]. A "chromatographic process in olfaction" has been proposed, in which the separation of odorants is based on their chemical properties and flow dynamics within the nose, combined with odorant affinity for the olfactory receptors [36,37]. Previous experiments showed that more water soluble odorants (those with a high sorption rate) are completely absorbed by the olfactory mucosa and removed from the air before they get to the ventral region of olfactory epithelium [36,38]. In addition, it is important to mention that fluid dynamic modeling showed higher air flow through the dorsal region [3,39,40]. Higher air flow permits odorants to distribute throughout the dorsal epithelium. Odorants with higher sorption rates could then activate receptors in this high air flow dorsal region, while odorants with lower sorption rates would not have time to interact with the receptors in that region. Consequently, odorants with a lower sorption rate will have more time to interact with the receptors that are located in the lower air flow ventral region, and higher sorption rate odorants might not even make it to the ventral zone due to complete absorption in the dorsal zone.

Based on these results it has been predicted that the strongly absorbed odorants (i.e. more water soluble) interact with dorsal region of the olfactory epithelium (zone I) and consequently activate the dorsal region of the olfactory bulb [3]. In turn, odorants with lower solubility have more time to reach the ventral regions of the olfactory epithelium, and subsequently lead to the activation of the ventral regions of the olfactory bulb [38,39,41]. The relationship between differences in odorant solubility and the topography of the projections from the epithelium to the olfactory bulb has been extensively studied [42-46]. Recently, Johnson et al. analyzed results of glomerular mapping from over 350 odorants and found that highly water-soluble odorants activated posterior regions of OB (halfway between dorsal and ventral extremes) [47]. These results are in contrast with a generalized notion that highly water soluble odorants are recognized by Class I ORs located in the dorsal region of the olfactory epithelium, which projects to the dorsal aspect of the bulb.

In order to study the relationship between odorant solubility and volatility properties with the topographical location of their cognate ORs within the olfactory epithelium, we implemented the novel method of membrane microtransplantation developed by Miledi [48]. Plasma membranes rich in ORs from either the dorsal or ventral regions of the olfactory epithelia were directly injected into X. laevis oocytes and tested against a panel of odorants with a broad range of solubility and volatility. Olfactory receptor activation was tested via electrophysiology, using the cystic fibrosis transmembrane regulator (CFTR) as a reporter [49].

## Methods

Mouse olfactory epithelium was obtained under a tissue sharing protocol approved by the University of Miami Internal Animal Care and Use Committee.

### O-MACS immunolabeling and two photon microscopy

Mice, strain C57BL, age 10-12 months, were sacrificed by CO_2 _asphyxiation for 2 min and by cervical dislocation. The skin was removed, and the head was split along the sagittal plane into left and right hemispheres. Each hemisphere was first fixed in 50 mL 4% paraformaldehyde (about 10× volume of the sample) in the 0.1 M phosphate buffer, pH 7.4 for 5 hours at room temperature. After fixation, each hemisphere was washed 3× for 15 min in TBS-T (50 mM Tris, 140 mM NaCl and 0.2% Triton X-100). Blocking was done in 10% NGS (Normal goat serum, Rockville) in TBS-T for 2 hours. Samples were washed 3× in TBS-T for 30 min. Primary anti-OMACS antibody (a kind gift from Dr. Hitoshi Sakano) was applied at 1:1000 dilution in blocking buffer and incubated for 16 hours at room temperature. Samples were again washed in TBS-T 3× for 30 min. Labeling was visualized using a fluorescent secondary antibody (goat anti-rabbit antibody conjugated to the HiLyte Fluor 488 fluorophore, AnaSpec #61056-H488). The secondary antibody was applied at 1 μg/mL in blocking buffer for 16 hours. We used Hoechst 33258 for nuclear staining at 10 μM. The images were obtained by two-photon microscopy (Zeiss/BioRad Radiance 2100 MP coupled with a Coherent Chameleon Ultra) of the intact olfactory epithelium at 955 nm excitation and using standard blue and green emission filter sets. Images are maximum Z-projections of 10-20 images at 5 micron steps. Each image is a Kalman average (n = 4) acquired at 16-bit resolution. Post-processing was accomplished with NIH ImageJ.

### Isolation of dorsal and ventral regions of olfactory epithelium

8 mice (C57BL/6J) 7.5 months old were killed by CO_2 _asphyxiation for 2 minutes and cervical dislocation. Mouse heads were separated in two halves by splitting along sagittal plane. Hemi-sections were put into ice-cold DMEM for 2 minutes and later immersed in ice cold PBS with protease inhibitors (100 μL PI/10 mL buffer, Sigma P2714). Both the dorsal and the ventral epithelia, and in some cases the respiratory epithelium, were carefully removed. Each part was gently blotted on the filter paper and immediately frozen in liquid N_2_. Results from previous experiments [16,50,51] have demonstrated bilaterally symmetric spatial pattern of ORs expression, thus allowing us to combine left and right hemi-sections. Tissue samples from 8 animals were pooled for the dorsal and ventral regions (totaling approximately 170 mg, 320 mg, respectively).

### Isolation of cilia

To detach cilia from dorsal and ventral pooled epithelium samples, we used a modified "calcium shock" method [52-54]. Pooled frozen samples from dorsal and ventral regions (isolated as discussed above) were placed into an ice-cold small Petri dish containing 1000 μL Buffer A, at pH 8.0 ("Buffer A": 30 mM Tris-HCl, 100 mM NaCl, 2 mM EDTA, 1 mM PMSF). We slowly added 10 μL of 1 M CaCl_2 _in 2 μL increments to give a final concentration of 10 mM CaCl_2 _while the solution was continuously stirred for 18 min at 4°C. Next, the solution containing the tissue was centrifuged for 10 min at 1500 g and at 4°C. The supernatant (cilia) was carefully removed and centrifuged at 12000 g for 10 min. The pellet containing cilia was re-suspended in the glycine buffer, pH 9.0 and stored in aliquots, for not more than a month at -70°C and used for the plasma membrane preparation.

### Plasma membrane preparation and microtransplantation

Isolated cilia from dorsal or ventral regions were resuspended in glycine buffer, pH 9.0, (200 mM glycine, 150 mM NaCl, 50 mM EDTA, 300 mM sucrose), gently homogenized (manually) and centrifuged at 9,500 g for 15 min at 4°C [48,55]. The supernatant was ultracentrifuged for 2 h at 100,000 g and the pellet was resuspended in 5 mM glycine. Protein concentration was measured using Bradford Coomasie blue assay (Pierce) and adjusted to 1 mg/mL. 50 nL was injected into X. oocytes. We injected (microtransplanted) dorsal preparations into 600 oocytes and ventral preparations into 800 oocytes.

### Preparation of oocytes and cRNA injection

We first injected cRNA for CFTR (a cAMP-activated Cl^- ^channel) and Gα_olf _as previously described [49]. The next day, when both CFTR and Gαolf proteins were expressed in the oocytes we microtransplanted ciliary plasma membranes from either dorsal or ventral regions into oocytes. The construct containing human M1 muscarinic G-protein coupled receptor was purchased from the UMR cDNA Resource Center (Missouri University of Science and Technology, Rolla, MO 65409).

### Electrophysiology

Our electrophysiological assay for olfactory receptor activation was accomplished as previously described [49]. 24 hours after microtransplantation and 48 hours after Gα_olf _and CFTR injection, we recorded responses. Odorants were selected with a broad range in volatility and solubility. All odorants and compounds (like GABA, isoproterenol and IBMX) were either from Sigma Aldrich (St. Louis, MO) or Fluka (Fluka Chemie AG, Switzerland). Odorants were first dissolved in DMSO to 1 M or 100 mM solution, and then diluted in regular buffer ND96 as described in [56] and applied for 15 sec. Odorants with lower solubility were heated at 37°C water bath for few minutes. Activation of ORs results in an inward current recorded in two-electrode voltage clamp mode using the OpusXpress 6000A (Molecular Devices). Each oocyte was also challenged with 1 mM IBMX to confirm successful expression of CFTR. Our control oocytes were injected with M1 receptor, CFTR and Gα_olf _and challenged with 10 μM Ach for 5 sec. All odorants used in our study were tested with these control oocytes to guard against false positives resulting from direct activation of the CFTR reporter or non-specific activation of the M1 receptor.

**Figure 1:**
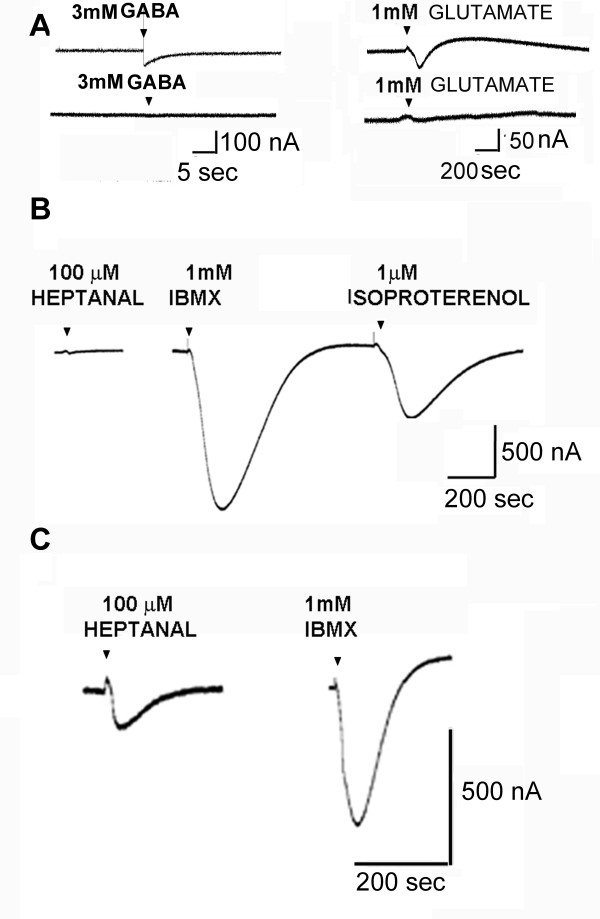
**Functional expression of receptors in X. oocytes microinjected with plasma membranes**. The current responses were recorded 24 hours after microtransplantation. **A**. GABA and glutamate responses in X. oocytes microinjected with mouse brain tissue. **B**. Responses from mouse respiratory epithelium to the odorant heptanal, the CFTR activator IBMX and the β2 adrenergic agonist, isoproterenol. **C**. Responses from mouse olfactory epithelial preparations to heptanal and IBMX. The response to 100 μM heptanal indicates specific responses from olfactory receptors, since it is absent from respiratory epithelium (B). Similar results were obtained from 10-12 oocytes tested.

For preliminary studies and method optimization, mouse olfactory and respiratory epithelium and brain were dissected, the tissue was homogenized and the plasma membrane preparations where isolated as described above, while skipping both ciliary membrane preparation step and the separation of dorsal and ventral parts (results shown in Figure 1). Oocytes expressing membranes from mouse brain were challenged with GABA to demonstrate successful microtransplantation of brain plasma membranes [57]. In addition, oocytes with brain preparations were challenged with 1 mM glutamate. Initially very small currents were observed; however after injecting mRNA for Gβ1 and Gγ3, responses were increased about 2.5 fold, thus indicating expression of metabotropic glutamate receptors. Constructs containing Gβ1 and Gγ3 subunits in the pcDNA3.1 vector were purchased from the UMR cDNA Resource Center [49]. Oocytes expressing membranes from the respiratory epithelium were challenged with isoproterenol to confirm successful microtransplantation of adrenergic GPCRs.

### Statistics

A two-tailed t-test was used to test for statistical significance (GraphPad Prism 5 software).

## Results

Our first goal was to verify the suitability of microtransplantation method. Plasma membranes from mouse brain were isolated and injected into oocytes. Current responses were recorded at a holding potential of -70 mV and 3 mM GABA was applied for 5 sec. The GABA response was fast; reflecting the activation of GABA-gated ion channels (Figure 1A, left panel). Control oocytes without microtransplanted plasma membranes challenged with 3 mM GABA did not produce any current (Figure 1A, right panel). Thus, we demonstrated expression of functional GABA_A _receptors from mouse brain via microtransplantation in Xenopus oocytes (Figure 1A) [57]. In addition, mouse brain membrane preparations injected in oocytes together with Gβ1 and Gγ3 were challenged with 1 mM glutamate. The representative trace is presented in the Figure 1A, right. No response was seen in un-injected oocytes when challenged with 1 mM glutamate (Figure 1A, right side, trace below).

The mouse olfactory epithelium is easily distinguishable by its yellow-brownish color and differs from the respiratory epithelium, which is mainly transparent and highly vascular. To confirm our ability to isolate the dorsal region of the olfactory epithelium, we used a dorsal zone marker, O-MACS. O-MACS immunostaining of the anterior, middle and posterior part of dorsal zone (yellow, zone I) is presented in the Additional file 1: Supplemental Figure S1.

In order to implement microtransplantation method to study dorsal and ventral ORs responses to different odorants, we injected plasma membranes from mouse respiratory and olfactory epithelium. When using the X. oocyte system for heterologous expression of olfactory receptors, the olfactory-specific signal transduction G-protein Gα_olf _and a reporter channel are required for the detection of functional ORs [49]. Plasma membrane preparations injected without Gα_olf _and CFTR yielded no responses when tested against 10 mixtures (each mixture contained 10 odorants, each at 300 μM, results not shown). This demonstrated the need for signal amplification (Gα_olf_) and the reporter channel (CFTR). Application of the odorant heptanal (100 μM) initiated a current response in oocytes injected with olfactory epithelium (Figure 1C), but not in oocytes injected with respiratory epithelium (Figure 1B). However, the isoproterenol (1 μM), the β2 adrenergic agonist, initiated a current response in oocytes injected with respiratory epithelium (but not olfactory epithelium), indicating successful and specific expression of membrane proteins [58,59]. The presence of heptanal responses in olfactory epithelium injected oocytes and their absence from respiratory epithelium injected oocytes demonstrates the specificity of the olfactory response and successful expression of olfactory receptors via microtransplantation approach.

We do believe that if indeed explicit representation of the location of olfactory receptors and their likely ligands/odorants exist in the olfactory system, we should have been able to detect it by studying these two regions with distinct anatomy and air flow dynamics.

We decided to isolate plasma membranes from cilia in combination with Gα_olf _and CFTR as signaling partners. Expression of functional ORs was studied using two-electrode voltage clamp against a panel of 100 odorants at 300 μM concentration. These odorants show a broad range in water solubility and volatility, expressed as log values in Additional file 2: Supplemental Table S1. In addition, molecular weight, formula, octanol/water partition coefficient (log P) and polar surface area (PSA) parameters for all odorants are presented. The water solubility of the selected odorants ranged over one million times, from 1.70 mg/L for farnesol (log solubility = 0.23, compound 98) to 1 × 10^6 ^mg/L for pyrrolidine (log solubility = 6, compound 50). Odorant volatility ranged over 10 billion times, from 1.07 × 10^-8 ^mmHg for nonanedioic acid (log volatility = -7.9, compound 51) to 538 mmHg for diethylether (log volatility = 2.73, compound 46).

**Figure 2:**
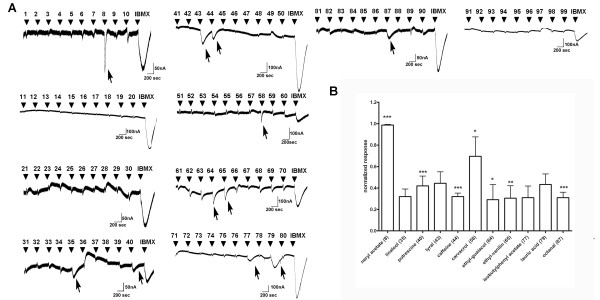
**Current responses to 100 odorants in oocytes injected with plasma membranes from the dorsal region of olfactory epithelium**. **A**. Representative traces. Odorants were applied at 300 μM, for 15 sec and at -70 mV holding potential. Odorant number is shown at the indicated time of application. At the end of the each run, 1 mM IBMX was applied for 5 sec. Arrows indicate responses. **B**. Current responses of dorsal region injected oocytes were normalized to the IBMX response in each oocyte (n = 3-8, mean ± SEM). Responses were normalized to the IBMX response in each oocyte. As a control, M1 receptor was injected and tested for the same odorants (n = 8, mean ± SEM). Significant differences when compared to M1 receptor responses were indicated as * for p ≤ 0.05, ** for p ≤ 0.001 and *** p ≤ 0.0001.

**Figure 3:**
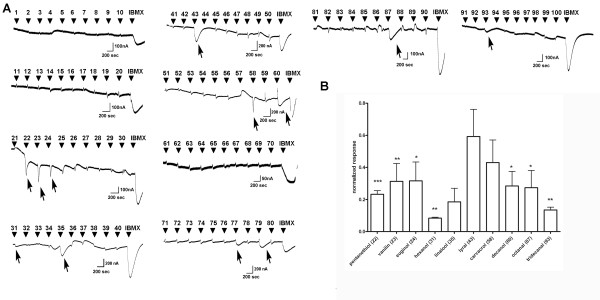
**Current responses to 100 odorants in oocytes injected with plasma membranes from ventral region, endoturbinates**. **A**. Representative traces. Odorants were applied at 300 μM, for 15 sec and at -70 mV holding potential. Odorant number is shown at the indicated time of application. At the end of the each run, 1 mM IBMX was applied for 5 sec. Arrows indicate responses. **B**. Current responses of ventral region were normalized to the IBMX response in each oocyte (n = 3-8, mean ± SEM). As a control, M1 receptor was injected and tested for the same odorants (n = 8, mean ± SEM). Significant differences when compared to M1 receptor responses were indicated as * for p ≤ 0.05, ** for p ≤ 0.001 and *** p ≤ 0.0001.

ORs microtransplanted from the dorsal region of mouse olfactory epithelium were challenged with a set of 10 odorants in a single run (odorants 1-10, 11-20 etc.). At the end of each run 1 mM IBMX was applied to verify expression of the reporter, CFTR. ORs from dorsal region responded to the following odorants: neryl acetate (compound 8 in Additional file 2: Supplemental Table S1), putrescine (compound 40), caffeine (compound 44), ethyl guaiacol (compound 64), ethyl vanillin (compound 65) and octanal (compound 87). All these responses were detected in 3-8 separate recordings. Representative traces are presented in Figure 2A. Summary of the results with significant differences in odorant responses evoked by receptors from dorsal regions were compared to the responses in control, M1 GPCR, Gα_olf _and CFTR expressing oocytes, and significant differences from M1, Gα_olf _and CFTR are indicated with * (Figure 3B).

In parallel experiments, ORs from the ventral region responded to: pentanethiol (compound 22), vanillin (compound 23), eugenol (compound 24) hexanol (compound 31), decanol (compound 60), octanal (compound 87) and tridecanal (compound 93). Representative traces are presented in Figure 3A. Significant differences in odorant responses evoked by receptors from ventral regions were compared to the responses in control, M1 GPCR, Gα_olf _and CFTR expressing oocytes and significant differences from M1, Gα_olf _and CFTR are indicated with * (Figure 3B).

**Figure 4:**
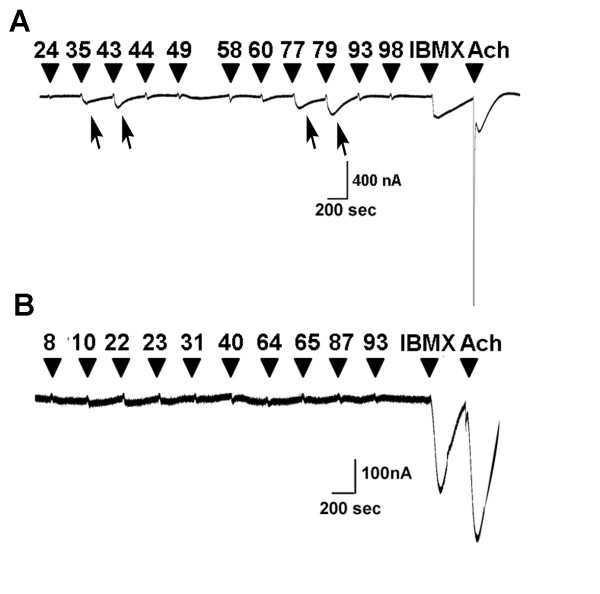
**Confirmation of OR specificity using M1 receptor as a control**. Representative traces to the selected odorants in X. oocytes injected with a control, M1 receptor (**A **and **B**). Odorant number is shown at the indicated time of application. At the end, 1 mM of IBMX and 10 μM of ACh was applied for 5 sec.

To control for non-specific responses, all odorants were tested in the M1 muscarinic receptor expressing oocytes. Responses in control oocytes expressing the M1 GPCR, along with Gα_olf _and CFTR to the odorants listed in the Figure 2 and 3 are presented in the Figure 4. Control oocytes injected with M1 muscarinic receptor, Gα_olf _and CFTR responded to the following odorants: linalool (compound 35), lyral (compound 43), isobutylphenyl acetate (compound 77) and lauric acid (compound 79); indicating direct CFTR activation, independent of olfactory receptor activation. CFTR is known to have many activators belonging to different chemical classes including flavones, xanthines, benzimidazoles, triazines and thiazolidine like compounds [60].

The physico-chemical properties of odorants activating olfactory receptors in dorsal and ventral regions of the olfactory epithelium were analyzed by hierarchical cluster analysis (UPGMA algorithm, MolSoft LLC.) Kurtz et al [61] showed that solubility of hydrophilic odorants in the mucosa can be predicted by their air/water partition coefficient. However, this cannot be applied to hydrophobic odorants, since they get dissolved in the lipophilic mucus and consequently show increased diffusion [61]. Thus, as an additional predictor of odorant solubility we used the octanol/water partition coefficient. Descriptors used in cluster analysis were: log of water solubility and volatility (data obtained from SRC, Syracuse Research Corporation), PSA -polar surface area and Log P were calculated by MolSoft software [62] (Additional file 2: Supplemental Table S1). Hierarchical clustering failed to detect separate clusters based on these odorant descriptors and differential odorant responses from dorsal and ventral region.

Despite the detection of different odorants by the two regions, the significant overlap of odorant properties made separation based on physico-chemical properties into clusters impossible.

Water solubility is described by log P - the octanol/water partition coefficient, where low water soluble compounds have log P ≥1, while highly water soluble compounds have log P ≤ -1. Plasma membrane preparations from dorsal region of olfactory epithelium responded to odorants that have log P in the range from -1.25-3.9, while preparations from ventral region responded predominantly to more hydrophobic compounds with log P ranging from 1.36-5.31. However, the difference in the mean log P value of odorants between these two membrane preparations was not significant (two-tailed t-test, P = 0.4507, confidence level α = 0.05) (Additional file 3: Supplemental Figure S2A). Our set of 100 odorants included some highly volatile odorants like hexane, acetone and diethyl-ether. We did not detect responses to these odorants in our plasma membrane preparations. The volatility of odorants detected by ORs from the ventral domain ranged from 1.18^-4 ^mmHg for vanilin, compound 23 (log volatility = -3.9) to 13.8 mmHg for 1-penthanethiol, compound 22 (log = 1.13). The volatility of odorants detected by ORs from the dorsal domain ranged from 1.04^-5 ^mmHg (ethyl vanilin, log = -4.983) to 41.2 mmHg (putrescine, log = 0.61). The difference in the mean volatility values of odorants between dorsal and ventral membrane preparations was not significant (t-test, two tailed, P = 0.929, confidence level α = 0.05) (Additional file 3: Supplemental Figure S2B). There were no significant differences in the mean water solubility (expressed as mg/L and presented as log values) of odorants detected from dorsal and ventral regions (t-test, two tailed, P = 0.248, at confidence level α = 0.05, Additional file 3: Supplemental Figure S2C).

## Discussion

Our ciliary plasma membrane preparations, enriched with olfactory receptors were isolated and fused with the X. oocyte plasma membrane by direct injection [63-66].There are many advantages of this microtransplantation approach [66] when compared to cRNA injection of known OR sequences. First, microtransplantation of ORs allows us to study receptors from defined regions within the olfactory epithelium (dorsal and ventral). The second advantage is that we can study a number of ORs simultaneously. Microtransplantation has also been successfully applied to study neurotransmitter receptors from postmortem brains in the context of Alzheimer's disease [57], autism [55] and epilepsy [67]. In the abovementioned references, receptors under study were ligand-gated ion-channels (e.g. ionotropic GABA and glutamate receptors) and the voltage-gated Ca^+2 ^and Na^+ ^channels.

We confirmed our ability to identify dorsal region using OMAS-immunolabeling (Additional file 1: Supplemental Figure S1). The precise function and significance of OMACS-exclusive expression in this region of olfactory epithelium remains unknown; however, few possible roles have been indicated. One possibility is that O-MACS activates medium chain fatty acids (which can be perceived as odorants) by addition of coenzyme A, thus producing acyl-CoA esters that are essential for many diverse metabolic processes including fatty acid synthesis, phospholipid synthesis and fatty acid oxidation. Another possibility is that O-MACS may have a role in zonal segregation of the OE during development, since its expression precedes OR expression [10].

This is a first time that the olfactory receptors have been successfully expressed using the microtransplantation approach. The total number of odorant responses from both dorsal and ventral regions was 14. This represents about 14% of all tested odorants probably reflecting the fact that by using microtransplantation approach, the responses we detect are from the most abundant receptors.

We detected octanal responses in both the dorsal and ventral regions of the olfactory epithelium (Figure 2 and 3). Recently, octanal was detected both as a volatile released from mouse body, as well as a significant component of mouse urine [68]. Our results confirm the importance of this aldehyde in the mouse world. In agreement with our results, Igarashi and Mori showed that octanal induced glomerular activity from both dorsal and ventral surface of rat olfactory bulb [69].

In this study, olfactory receptor proteins were microtransplanted with other membrane proteins from the olfactory epithelium in their "microenvironment", thus our system does not mirror heterologous expression in X. oocytes where cRNA of particular OR was injected. As mentioned earlier, we used 300 μM concentration of odorants. This value is high when compared with the odorant concentration used to elicit responses in glomeruli during in vivo experiments [70,71]. As summarized and explained by Oka et al. odorant sensitivity when tested in isolated olfactory sensory neurons or in the heterologous expression system is much lower than the sensitivity seen in the in vivo experiments in the olfactory bulb, due to the absence of factors like olfactory mucosa and air-flow dynamics [72].

We did not find evidence supporting correlation between the solubility and volatility of odorants and the functional expression of olfactory receptors in the dorsal or ventral region of the olfactory epithelia. Thus, no simple clustering or relationship between these parameters could be associated with the different regions. Odorants detected by ORs from the dorsal and ventral regions showed overlap both in volatility and water solubility, indicating that the location of the ORs within olfactory epithelium is not related to the physicochemical properties.

Odor coding is a result of the interplay of many different factors, and we are just beginning to understand the role of some of them. For example, as discussed in Scott et al. paper [34] an increase in carbon chain length (in case of hydrocarbons) correlates with the responses in the ventral region. In addition, the airflow pattern along the nasal cavity has a significant effect on odor coding [73] as well as selective projection of axons related to the particular olfactory receptor to specific glomeruli [74].

A comprehensive review of current knowledge regarding spatial organization of the odorant receptor maps was presented by Mori et al. [75], while Johnson and Leon recently summarized the importance of the chemotopic odorant coding in olfactory perception [76]. Mori et al. summarized their previous work and mapped glomerular responses to 72 odorants divided into 12 structural classes in the dorsal surface of an olfactory bulb. We used 18 compounds that were the same (from 10 structural groups listed in Mori's paper, the numbers in parenthesis are numbers of volatile compounds from Additional file 2: Supplementary Table 1): aliphatic ketones (compound 72), cyclic ketones (compounds 4 and 15), hydrocarbons (compound 5), cyclic alcohols (compound 7), aldehydes (compounds 10, 17 and 29), aliphatic alcohols (compounds 12, 26, 31 and 60), aromatic aliphatic ketones (compound 14), phenyl ethers (compounds 1, 16 and 24), phenols (compound 37), aliphatic acids (compounds 30 and 32). Two other structural groups were diketones and ethers. We used different representative of diketones: butanedione (compound 3) and different representative of ethers: diethyl ether (compound 46).

In summary, all the structural classes in Mori et al. are represented in our paper, however some compounds are different. We do want to point out that our primary reason in selection of volatile compounds was their broad range in volatility and solubility. In contrast to this previous work, we directly evaluated the hypothesis that olfactory receptors are spatially organized within the olfactory epithelium based on the chemical properties of their ligands.

We compared the volatility and water solubility parameters for odorants that activate rat I7 olfactory receptor (including C7-C11 aldehydes, trans-2-octenal, citral and citronellal [77]) and found that water solubility among these odorants differed over a 67-fold range (20.2 for undecanal to 1340 mg/L for citral). Volatility differed 57-fold, (0.06 for undecanal to 3.52 mmHg for heptanal). Compared to the ranges detected in our study, these results indicate a relatively small range in volatility and solubility of the preferred ligands for this particular receptor. It would be interesting to see whether this applies to other olfactory receptors, particularly those considered broadly tuned. Whether our results, showing the broader range in odorants volatility from dorsal region (over 3,900 000 times compared to 100 000 times from ventral region) are consequence of increased diversity of ORs in that region or are the result of the presence of more broadly tuned receptors, remains to be tested.

Because each odorant is delivered to the oocyte in an identical fashion, the different kinetics (rise time, decay time, and width) of responses evoked by different odorants both in dorsal and ventral regions may indicate different receptor density, sensitivity and/or different modes of desensitization (see traces, Figures 3 and 4). A plethora of GPCR signaling components is known to be endogenously expressed in X. oocytes [78-81]. A recent study by the Lefkowitz group [82] demonstrated ligand bias towards different desensitizing pathways. This offers an attractive explanation for the variability of the kinetics of the responses in Figures 3 and 4. It would be interesting to study whether similar mechanisms exist in olfactory sensory neurons. This would add an additional dimension in our understanding of activation/de-activation (desensitization) of olfactory receptors. A huge odorant space, a huge number of olfactory receptors and now the possibility that the odorants/ligands of the same receptor differentially desensitize the receptor, evokes even more complexity not just in the temporal dimension of odorant detection, but also in the whole process of olfactory perception.

## Conclusions

We did not detect a significant correlation between the physicochemical properties of odorants (solubility and volatility) and the functional expression of olfactory receptors between the dorsal and the ventral region of the olfactory epithelia. A differential sorption of odorants in the mouse peripheral olfactory system is likely to be mediated by air flow dynamics and physico-chemical properties of olfactory mucosa.

## Competing interests

The authors declare that they have no competing interests.

## Authors' contributions

TA conceived and designed the study, set up the experiments, performed immunostaining, analyzed the data and wrote the paper. RAD performed microscopy and critically revised the manuscript. Authors read and approved the final manuscript.

## Supplementary Material

Additional file 1 **Supplemental Figure S1: Scheme of mouse olfactory epithelium and dorsal immunolabeling**. **A**. OMACS immunolabeling of the dorsal region (green). Blue indicates nuclear staining. The images were obtained by two-photon microscopy (Zeiss/BioRad Radiance 2100MP coupled with a Coherent Chameleon Ultra) of the intact olfactory epithelium at 955 nm excitation and using standard blue and green emission filter sets. Images are maximum Z-projections of 10-20 images at 5 micron steps. Each image is a Kalman average (n = 4) acquired at 16-bit resolution. Post-processing was accomplished with NIH ImageJ. **B**. The scheme of the sagittal view of the left hemisphere of mouse olfactory epithelium. A dorsal region, zone I is colored in yellow and ventral region (endoturbinates (IIa, IIb, III and IV) in orange. OB-olfactory bulb. Dashed lines indicate sites of immunostaining images of anterior (A), middle (M) and posterior (P) part of dorsal region (**A**). Click here for file

Additional file 2 Supplemental Table S1: 100 odorants with structures and all physico-chemical parameters used in Cluster analysis. Click here for file

Additional file 3 **Supplemental Figure S2: Overlapping physico-chemical properties of odorants detected by dorsal and ventral region. A**. log P (octanol/water partition coefficient) **B**. log volatility (mmHg) **C**. log water solubility (mg/L) and **D**. log PSA (polar surface area in Å^2^). Dotted lines indicate the range in log P, water solubility, volatility and PSA of all 100 odorants used in the experiment. Each dot represents a single physico-chemical value from each odorant that evoked responses from either dorsal or ventral region. Click here for file
