# Supplementary figures and images for: The location of olfactory receptors within olfactory epithelium is independent of odorant volatility and solubility
Source: BMC Res Notes. 2011 May 6;4:137. doi: 10.1186/1756-0500-4-137 (PMC3118157; doi:10.1186/1756-0500-4-137)

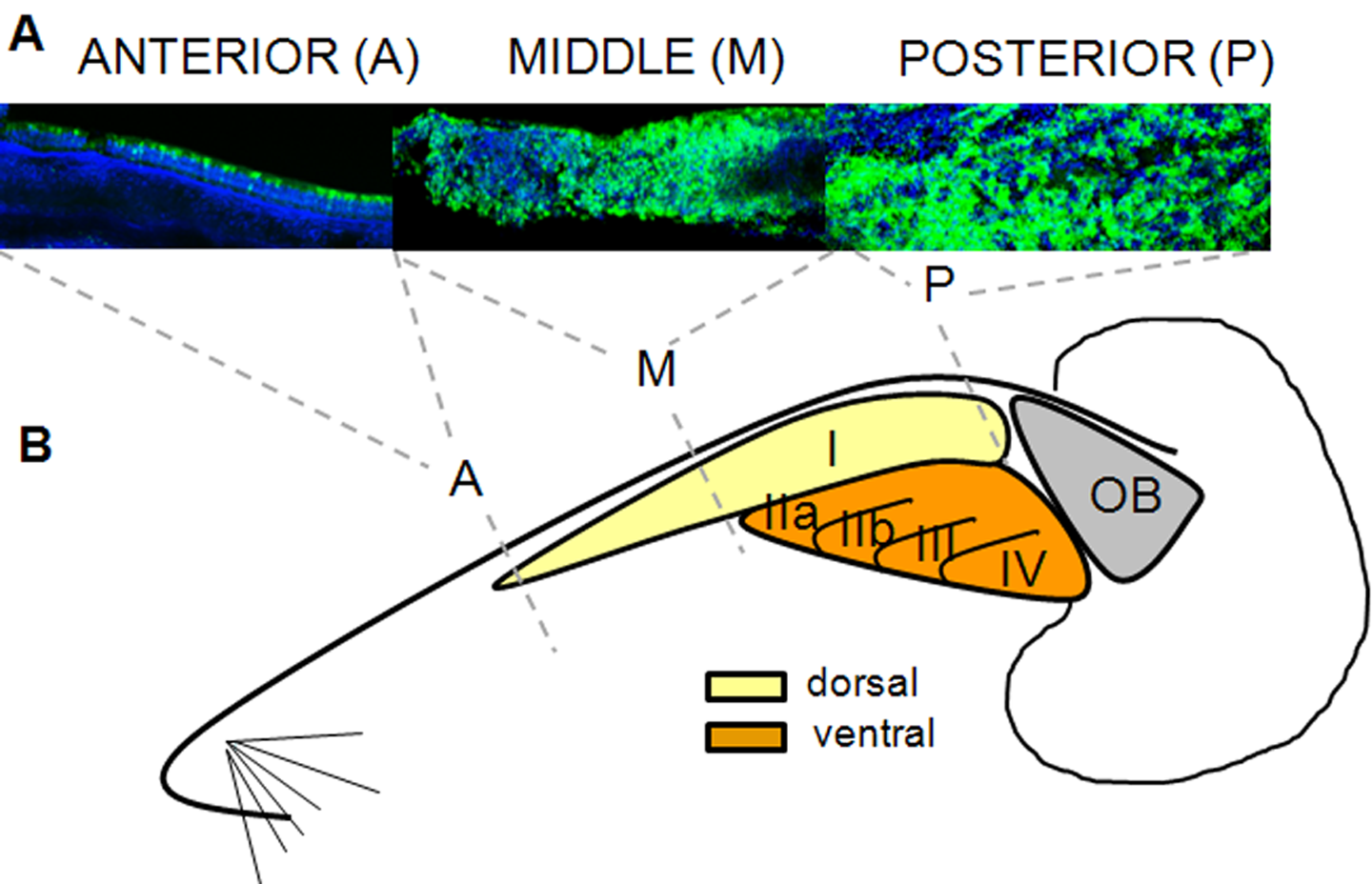

Supplement: Additional file 1 — Supplemental Figure S1: Scheme of mouse olfactory epithelium and dorsal immunolabeling. A. OMACS immunolabeling of the dorsal region (green). Blue indicates nuclear staining. The images were obtained by two-photon microscopy (Zeiss/BioRad Radiance 2100MP coupled with a Coherent Chameleon Ultra) of the intact olfactory epithelium at 955 nm excitation and using standard blue and green emission filter sets. Images are maximum Z-projections of 10-20 images at 5 micron steps. Each image is a Kalman average (n = 4) acquired at 16-bit resolution. Post-processing was accomplished with NIH ImageJ. B. The scheme of the sagittal view of the left hemisphere of mouse olfactory epithelium. A dorsal region, zone I is colored in yellow and ventral region (endoturbinates (IIa, IIb, III and IV) in orange. OB-olfactory bulb. Dashed lines indicate sites of immunostaining images of anterior (A), middle (M) and posterior (P) part of dorsal region (A). [file 1756-0500-4-137-S1.TIFF]

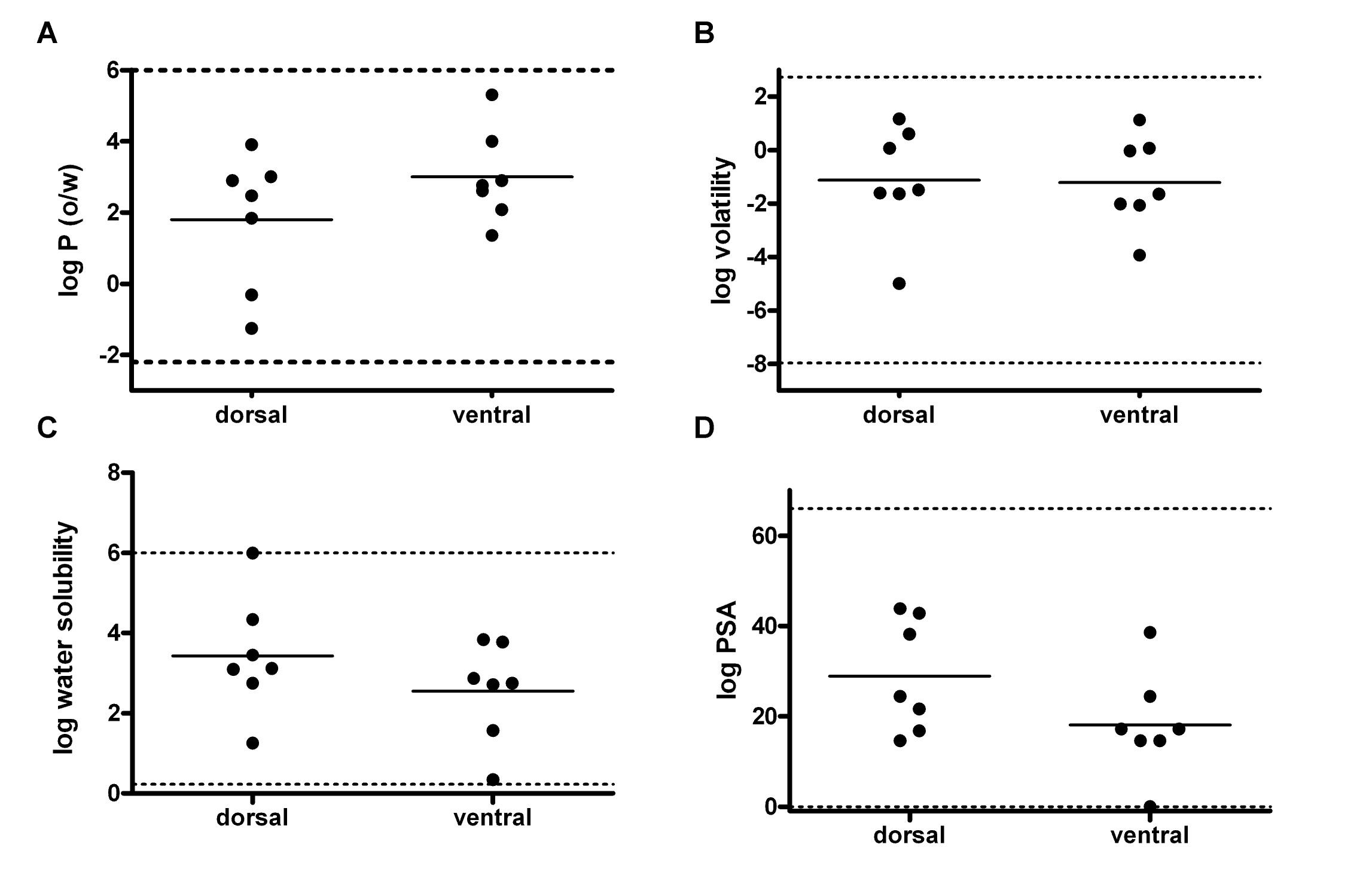

Supplement: Additional file 3 — Supplemental Figure S2: Overlapping physico-chemical properties of odorants detected by dorsal and ventral region. A. log P (octanol/water partition coefficient) B. log volatility (mmHg) C. log water solubility (mg/L) and D. log PSA (polar surface area in Å2). Dotted lines indicate the range in log P, water solubility, volatility and PSA of all 100 odorants used in the experiment. Each dot represents a single physico-chemical value from each odorant that evoked responses from either dorsal or ventral region. [file 1756-0500-4-137-S3.TIFF]
